# From rugby to basketball: a comparative analysis on the implementation of mixed ability

**DOI:** 10.3389/fspor.2026.1769269

**Published:** 2026-03-16

**Authors:** Pablo Elipe-Lorenzo, Carla da-Silva, Pelayo Diez-Fernández, Brais Ruibal-Lista, Miguel Saavedra-García, Sergio López-García

**Affiliations:** 1 Faculty of Education, Universidad Pontificia de Salamanca, Salamanca, Spain; 2 Research Group in Physical Activity, Sport and Health (GIADES), Faculty of Education, Universidad Pontificia de Salamanca, Salamanca, Spain; 3 Research Group in Sport Sciences (INCIDE), Department of Physical Education, Universidade da Coruña, A Coruña, Spain; 4 International Mixed Ability Sports Spain (IMAS Spain), Vitoria-Gasteiz, Spain; 5 Department of Functional Biology, Universidad de Oviedo, Oviedo, Spain; 6 EUM Fray Luis de León, Universidad Católica de Ávila, Valladolid, Spain

**Keywords:** disability, inclusive communities, inclusive sport, quality of life, social exclusion

## Abstract

**Introduction:**

People with disabilities continued to face numerous barriers to participation in mainstream sports clubs. In this regard, the Mixed Ability (MA) model emerged as a response to the limited availability of inclusive opportunities that enabled involvement in these contexts and, consequently, the exercise of individual rights. Therefore, the aim of this study was to analyse the application of the MA model in basketball and to compare it with rugby, a discipline with a more consolidated trajectory in this field. Furthermore, it sought to assess how participation influenced the perception of disability among the different stakeholders involved.

**Methods:**

A quantitative cross-sectional design with descriptive and relational components was applied. The Q-NeMAR scale was used as the assessment instrument. In total, 123 individuals (59 men and 63 women) from basketball and 114 individuals (74 men and 39 women) from rugby participated.

**Results:**

The results revealed consistently high ratings across all analysed dimensions. Significant differences were observed between sports in items related to visibility, promotion of specific plans, training, and intra- and interpersonal factors, with a more favourable perception among the rugby group. In addition, most participants reported understanding disability from a social and rights-based perspective.

**Discussion:**

These findings suggested that participation in MA settings fostered a shift from assistentialist conceptions towards models of active citizenship. Overall, the results reflected a broadly positive perception of the consolidation, promotion, and sustainability of the MA model as a pathway for inclusion and sporting participation.

## 1 Introduction

The World Report on Disability estimated that over 1.3 billion people live with some form of disability, representing about 16% of the global population, and this number continues to rise worldwide (1). The United Nations (UN) 2030 Agenda stresses the need to reduce inequalities within the Sustainable Development Goals (SDGs), while the Convention on the Rights of Persons with Disabilities (CRPD) (2) establishes full and effective social participation as a fundamental principle. In Spain, the national legal framework—Royal Legislative Decree 1/2013 on the rights and social inclusion of persons with disabilities and Law 39/2022 on Sport—reinforces social cohesion and equality (3, 4). The latter specifically promotes inclusive sport as a strategy to foster shared activities between people with and without disabilities on equal terms.

Within this context, it was essential to reflect on what inclusion truly meant within sport clubs, as academic literature had identified multiple interpretations of this concept. Jeanes et al. (5) highlighted that, in many cases, so-called “inclusive” sections operated independently from the rest of the club, with little interaction between their members and other sections. True inclusion in sport, therefore, was not limited to ensuring the presence of persons with disabilities (PwD), but rather involved offering them the opportunity to decide how and with whom they wished to participate (6). Inclusion, by contrast, encompasses fully open activities in which people with and without disabilities participate together under standard rules, with minimal or no adaptations, promoting equal participation for all (7). Pearce and Sanderson (8) argued that adapting sporting rules without considering the personal preferences of PwD could result in merely superficial inclusion, where neither social nor personal development was fully achieved. Likewise, social norms influenced the degree to which PwD were able to exercise self-determination in accessing and participating in sporting activities, particularly depending on how their competence, autonomy, and sense of belonging were perceived. From this perspective, ableism could directly limit such self-determination, and the social dynamics within this context tended to reproduce and reinforce ableist attitudes (9). To achieve genuine and effective inclusion, PwD had to be not only part of these spaces but also play an active and meaningful role in sporting activities (10). Corazza and Dyer (11) pointed out that social inclusion was not a static state but a dynamic process shaped by the relationship between the individual and their environment, as well as by their interpersonal connections and community engagement. Community participation is widely regarded as a fundamental component of human functioning and a key factor influencing the quality of life of PwD (12, 13).

At present, an emerging international paradigm integrates quality of life principles with the systems-of-supports approach, giving rise to what has been termed the Quality of Life Supports Paradigm (QOLSP) (14). This integrative framework is grounded in the social model of disability, which conceptualises disability as the result of interactions between individuals and their environments (15, 16), and is firmly rooted in a person-centred approach that prioritises individual preferences, goals, and life projects (17). Thus, the QOLSP seeks to enhance opportunities for PwD to access and meaningfully engage with people, settings, and activities they genuinely value, as well as to define the lives they wish to lead and the environments in which they choose to participate (16). Thus, genuine inclusion depended on promoting participatory and authentic environments (18). Along these lines, PwD tended to prefer integration within inclusive community sport clubs, avoiding segregated environments and seeking shared participation spaces (19). Nevertheless, they still faced numerous barriers to participating in mainstream clubs on equal terms without classification systems or specific adaptations (20). In Spain, the development of community-based services remained limited (21). Consequently, although the freedom of choice proposed by Misener and Darcy (6) might have existed in theory, the lack of genuinely inclusive opportunities constrained the full exercise of self-determination (22).

Against this background, the Mixed Ability (MA) model emerged as a rights-based and community-driven approach to inclusive sport, originally developed by PwD to adress the lack of inclusive opportunities to participate in full-contact rugby within mainstream clubs in their communities. Later, the International Mixed Ability Sports (IMAS) organisation institutionalised this initiative, transforming it into a social movement promoting inclusion within mainstream sport. IMAS supports grassroots clubs in establishing MA teams and co-produces educational resources in collaboration with participants. Through this community-based and participatory approach, IMAS aims to promote sustainable engagement in sport, reduce social exclusion, and dismantle barriers between participants with and without disabilities, thereby fostering long-term social change (11, 23). The model has expanded across multiple sports. In Spain, it is present only in rugby and basketball, while internationally it includes cricket, floor curling, football, golf, tennis, and boxing, among others. In Spain, rugby stands out as the most established MA sport, with the highest number of active teams (24).

The MA model is grounded in the principle that players with and without disabilities participate together on equal terms, without classification systems or segregated structures. Sporting activities are governed by the standard rules of each discipline without formal adaptations, allowing only minor, individualised adjustments when necessary to support participation. This approach challenges traditional deficit-based perspectives by emphasising shared participation and recognising diversity as a normative characteristic of all players (11). A central feature of the model is the rejection of hierarchical roles commonly found in disability sport. Players without disabilities are explicitly positioned as teammates rather than helpers or volunteers, fostering reciprocal relationships and dismantling the traditional “us and them” dichotomy (23). Teams are fully integrated within existing sports clubs as one additional section, promoting shared identity, visibility, and social interaction beyond the playing field (11). Research showed that implementing the MA model was an effective pedagogical tool for fostering genuine inclusion (25, 26). Furthermore, Dyer and Sandford (27) reported that many sports clubs became aware of entrenched exclusionary practices when they engaged directly with disability through participation in MA. In this sense, when equal participation is achieved, it extends beyond the normative aim of recognition justice and produces benefits for everyone involved (23).

**Figure 1:**
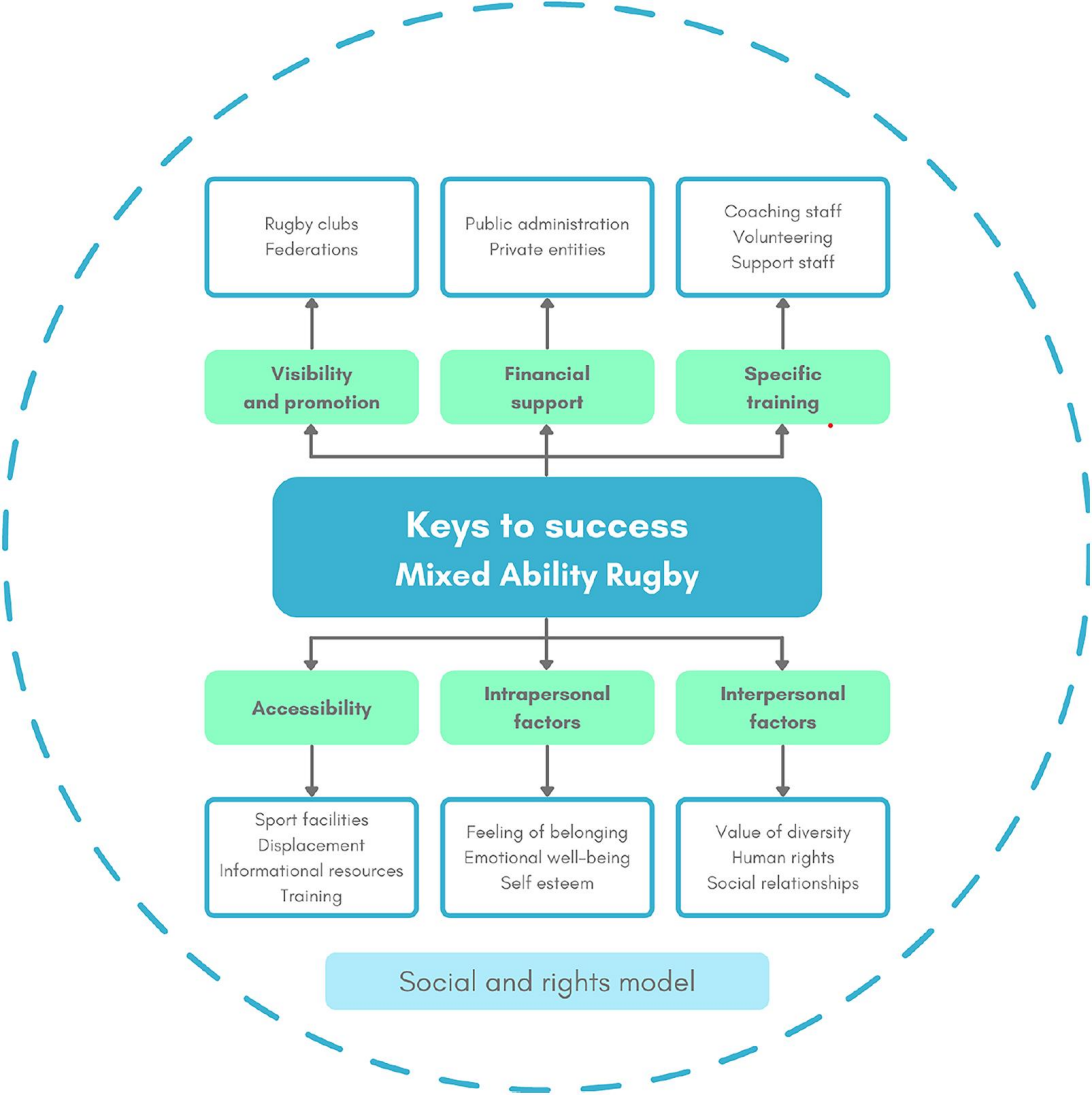
Framework of the keys to success in Mixed Ability rugby. From da-Silva (22).

From a conceptual perspective, its effectiveness relies on the interaction between organisational support (e.g., funding, institutional backing), social factors (e.g., team culture, peer relationships), and personal experiences (e.g., self-determination, sense of belonging), all framed within a rights-based and equity-oriented approach (22). Figure 1 illustrates these key components and their role in supporting the holistic development of MA sport. Similarly, Elipe-Lorenzo et al. (28) examined the influence of the model on the quality of life of people with intellectual disabilities, reporting that longer participation in mainstream sport was associated with higher perceived quality of life, including among persons with moderate and severe intellectual disabilities. Qualitatively, participants emphasised key aspects of MA, such as its inclusive nature, the development of close and supportive relationships, enjoyment of participation, a strong sense of community, active involvement, and the opportunity to engage in new and meaningful experiences.

However, research on the MA model remained scarce, and in Spain, the limited available evidence had been developed primarily in the context of rugby (22, 28). Furthermore, to date, no empirical studies have examined the application of the MA model in basketball, nor have comparative analyses across different sports been conducted. Accordingly, the present study aimed to analyse the implementation of the MA model in basketball, comparing it with the established rugby experience, and to evaluate how participation influenced the perception of disability among the various stakeholders involved.

## 2 Methodolody

### 2.1 Type of study and design

This study was conducted within the framework of a quantitative, cross-sectional investigation carried out through a single data collection process. A descriptive and relational design was employed with the aim of characterising perceptions of disability within the context of MA basketball and exploring their relationship with sociodemographic and sporting experience variables.

The Baskonia Club represented the only reference in Spain that developed MA basketball and concentrated the largest number of active participants in this modality. For this reason, a non-probabilistic convenience sample was used, consisting of voluntary participants, as population accessibility made it impossible to apply probabilistic sampling.

### 2.2 Procedure

Initial contact with the Baskonia Club was established via email through the mediation of IMAS. The selection of this club was based on its pioneering role in MA basketball in Spain and the fact that it hosted the largest number of active participants in this field. Participants were selected according to the following inclusion criteria: being over 18 years of age and being identified as a player, family member or assistant, legal representative, coaching staff, support or management personnel, and referee from the MA basketball environment. No formal exclusion criteria were established beyond not meeting the inclusion requirements; however, individuals who did not provide informed consent were excluded from the final sample.

Data were collected by the first researcher, who had prior experience in MA contexts. The researcher stayed in the city of Vitoria-Gasteiz for a four-week period, from 24 October to 20 November 2024, regularly attending team training sessions to administer a paper-based questionnaire. The Baskonia Club had two men's teams and one women's team, and one weekly training session per team was used to distribute the questionnaires to players, coaches, and to family members, assistants, or legal representatives, who participated voluntarily without financial compensation. For referees and management staff, due to the difficulty of arranging a simultaneous in-person meeting, the questionnaire was distributed in an online format. To promote comprehension and accessibility, questionnaires were provided in two versions: an original version and an easy-to-read version, to ensure self-determination and equitable participation. In line with previous recommendations, additional supports were offered to facilitate understanding beyond the easy-to-read format alone (29).

Moreover, to minimise potential social desirability bias, participants were assured that their responses would remain anonymous and confidential, and that data would be used exclusively for research purposes. Questionnaires were returned in sealed envelopes to prevent coaches, teammates, or relatives from accessing individual responses. Additionally, participants were explicitly informed that the researchers responsible for data collection were not members of the club or its organisational structure, in order to foster trust and reduce any perception that their responses might influence their position within the sporting environment.

### 2.3 Participants

To conduct the comparative analyses proposed in the study objectives, data from MA rugby participants were also included. These data were drawn from the database compiled by da-Silva et al. (30), who granted permission for their use in this research. A total of 237 individuals participated in the study, including 114 participants from MA rugby and 123 from MA basketball. Table 1 presents the sociodemographic characteristics of the sample across both sports. Overall, the samples were heterogeneous in terms of gender, roles within the clubs, and years of experience in MA sport. In rugby, a higher proportion of men participated, whereas the basketball sample showed a more balanced gender distribution. Participants were primarily based in Spain; however, in the rugby study conducted by da-Silva (22), the questionnaire was distributed to organisations listed in the IMAS database in Spain, Argentina, Chile, and Ecuador, which subsequently disseminated it within their respective clubs. Regarding roles, players, family members, technical staff, and referees were represented in both sports, reflecting the diversity of stakeholders involved in the MA environment. Most participants in both contexts reported between one and five years of experience in Mixed Ability sport. The mean age was comparable across sports, with values of 43.36% years in rugby, ranging from 18 to 65 and 44.0% years in basketball, ranging from 18 to 66 (Table 1).

**Table 1:** Sociodemographic characteristics of participants in MA rugby and basketball.

Variable	Category	Rugby (%)	Basketball (%)
Gender	Men	64.9	48
	Women	34.2	51.2
	Other	0.9	0.8
Country of participation	Spain	67.5	100
	Argentina	18.4	0
	Ecuador	8.8	0
	Chile	5.3	0
Role	Player	34.2	39
	Family member	26.3	40.7
	Technical staff	32.5	14.6
	Referee	7	5.7
Years of experience	<1 year	15.8	17.2
	1 to 3 years	36.8	29.5
	3 to 5 years	28.1	36.1
	5 to 7 years	13.2	17.3
	>7 years	6.1	0
Age	Mean	43.36	44.0

Table adapted from da-Silva (22). Author's elaboration.

### 2.4 Instrument

The present study used the Questionnaire to Identify the Needs Detected by the Mixed Ability Rugby Environment (Q-NeMAR), originally designed for the rugby context (22, 30). The Q-NeMAR was developed to identify needs, supports, and perceived barriers in inclusive sports environments following the MA model, evaluating dimensions such as material resources, social and technical support, structural barriers, learning outcomes, and social inclusion. In addition to the standard version, the instrument is also available in an easy-to-read format to facilitate accessibility and comprehension for participants with diverse cognitive abilities. The final version of the Q-NeMAR comprises 192 items organised into common sections (A–D) and specific sections (E1–E4). The common sections include: Section A, addressing the concept of disability; Section B, focusing on the MA concept; Section C, examining the needs associated with MA sport; and Section D, covering sociodemographic factors. The specific sections are tailored to different stakeholder groups: Section E1 for players; Section E2 for family members, personal assistants, support persons or legal representatives; Section E3 for coaches and club technical and support staff; and Section E4 for referees.

The validation of the instrument was obtained following a Delphi process and was supported by high levels of expert consensus in the final consultation round. In the third round (R3), the expert panel (*N* = 17) demonstrated a very high degree of agreement regarding the adequacy of the proposed items. The overall mean score of the questionnaire was over 5 points, with a very low standard deviation (SD = 0.113) and an average coefficient of variation of 2.30%, indicating strong consensus among experts. Only four items (2.08%) scored below the predefined adequacy threshold (mean < 4.50); however, all were retained due to their conceptual relevance to the MA model. Across all sections, mean item scores ranged from 4.82 to 4.99, reflecting a consistently high level of expert agreement. Most items are formulated using a 5-point Likert-type scale to assess the degree of adequacy or agreement with statements related to material resources, social and technical support, organisational aspects, learning outcomes, and social inclusion within MA sport environments. The internal consistency of the instrument was high. Cronbach's alpha was 0.901 for Section E1, 0.880 for Section E2, 0.942 for Section E3, and 0.956 for Section E4. Construct validity was supported by an acceptable level of sampling adequacy (Kaiser–Meyer–Olkin index = 0.677). Furthermore, Bartlett's test of sphericity confirmed the suitability of the data for factor analysis (*χ*^2^ (18,302) = 5,621.532; *p* < 0.01), indicating that the analysis of the questionnaire's dimensional structure was appropriate.

The adaptation process to basketball followed strict methodological standards and involved an expert review committee composed of two quantitative research methodologists and three specialists in the MA model. This process was carried out for both the standard and the easy-to-read versions of the questionnaire to ensure semantic equivalence, accessibility, and consistency across formats. The adaptation included an exhaustive review of items to ensure semantic relevance, cultural appropriateness, and content validity, while maintaining the conceptual structure of the original questionnaire. The process was carried out in collaboration with IMAS, which provided practical expertise and external validation from the professional basketball setting. The final version of the instrument retained the original 5-point Likert structure to assess the degree of agreement or frequency of perceptions, with an estimated completion time of 10–15 min.

### 2.5 Data analysis

Data were analysed using SPSS version 31, under an institutional licence provided by the Universidad Pontificia de Salamanca (UPSA). First, descriptive analyses appropriate to the nature of each variable were performed. For quantitative ratio-scale variables, mean, standard deviation, minimum, and maximum values were calculated. For qualitative ordinal variables, median, mode, and quartiles were reported. The internal consistency of the instrument was assessed using Cronbach's alpha, showing high reliability across all sections (E1 = 0.918, E2 = 0.963, E3 = 0.942, and E4 = 0.967).

Additionally, to assess associations between categorical variables, contingency tables and measures of association (phi and Cramer's V) were used, with Phi coefficients applied to dichotomous nominal variables and Cramer's V to polytomous nominal variables. These indicators were used to assess the strength of the relationship between previous experience in the MA model and perceptions of disability.

A significance level of *p* < .05 was adopted for all procedures. Furthermore, in addition to statistical significance, the interpretation of results prioritised effect size and practical relevance.

### 2.6 Ethical approval

The study was approved by the Ethics Committee of the UPSA (Record No. 14/06/2024) and was conducted in accordance with the recommendations of the Declaration of Helsinki. Ethical principles were ensured throughout the research by maintaining participant anonymity and the confidentiality of all information collected through questionnaires.

## 3 Results

The study revealed consistently high ratings among participants regarding structural, educational, and relational factors that influenced the development of MA basketball, with mean scores across all sections exceeding 4 out of 5 points (Table 2). The Mann–Whitney *U* test identified significant differences between basketball and rugby in several items (Table 3).

**Table 2:** Descriptive statistics by thematic blocks of mixed ability basketball and rugby.

Thematic Block	Items	Sport	M	SD	Minimum	Maximum	*N*
Visibility	5a–5g	Baketball	4.49	0.88	1	5	123
		Rugby	4.69	0.66			114
Promotion of Specific Plans	6a–6g	Baketball	4.40	0.96	1	5	123
		Rugby	4.57	0.78			114
Financial Support	7a–7g	Baketball	4.40	0.97	1	5	123
		Rugby	4.39	0.91			114
Training	8a–8g	Baketball	4.39	0.98	1	5	123
		Rugby	4.65	0.67			114
Accessibility	9a–9e	Baketball	4.57	0.84	1	5	123
		Rugby	4.59	0.75			114
Intrapersonal Factors	10a–10i	Baketball	4.54	0.82	1	5	123
			4.73	0.55			
		Rugby					114
Interpersonal Factors	11a–11h	Baketball	4.38	0.90	1	5	123
		Rugby	4.70	0.57			114

Table adapted from da-Silva (22). M = Mean; SD = Standard deviation. Author's elaboration.

**Table 3:** Mann–Whitney *U* test results between basketball and rugby for the different thematic blocks.

Variable/Item	Mean Rank Basketball	Mean Rank Rugby	Mann–Whitney U	*Z*	*p*
Visibility—Sports Clubs (5c)	108,84	129,96	5761,5	−3,439	.001
Visibility—Formal Education (5e)	112,14	126,40	6167,5	−2,013	.044
Promotion of Specific Plans—Sports Clubs (6c)	110,37	128,32	5949,0	−2,788	.005
Training—Coaching Staff (8b)	111,67	126,91	6109,5	−2,384	.017
Training—Referees (8c)	109,01	128,66	5796,0	−2.765	.006
Training—Support Staff (8f)	112,13	126,41	6166,0	−2,172	.030
Sense of Belonging to the Club (10a)	105,63	133,43	5366,0	−4,204	.000
Support from Other Categories (10b)	111,18	127,44	6049,0	−2.327	.020
Improvement in Self-Esteem (10e)	112,26	126,27	6182,0	−2,266	.023
Opportunity to Participate in Decision-Making (10i)	109,80	128,92	5880,0	−3.067	.002
Sports Insurance (11a)	106,33	132,67	5452,5	−3,811	.000
Federation Licence (11b)	108,43	130,41	5710,5	−2,696	.007
Membership Fee (11c)	97,19	142,53	4328,5	−5,354	.000
Trust Between Athletes and Club (11d)	110,62	128,04	5980,0	−2,710	.007

Table adapted from da-Silva (22). Author's elaboration.

Participants strongly agreed on the importance of visibility as a key element in MA basketball, with mean scores that ranged from 4.30 (SD = 1.02) to 4.59 (SD = 0.79). They highlighted the role of federations, clubs, educational institutions, and disability organisations in promoting dissemination through social media and digital platforms. Significant differences were found in visibility promoted by sports clubs (Z = –3.439, *p* = .001) and the education system (Z = –2.013, *p* = .044), with rugby participants assigning greater importance. Promotion-related items also showed high scores (M = 4.15–4.54), reflecting expectations for institutional strategies that ensured long-term sustainability. Only one significant difference appeared in promotion within the educational sphere (Z = –2.788, *p* = .005), again higher in rugby. Financial support was positively evaluated (M = 3.98–4.58), underlining consensus on the need for sustainable funding through grants and sponsorships, without significant differences between sports. Training among stakeholders was highly rated (M = 4.25–4.54). Significant differences were found in formal education for coaches (Z = –2.384, *p* = .017), referees (Z = –2.765, *p* = .006), and volunteers (Z = –2.172, *p* = .030), with rugby showing greater perceived needs. Accessibility was almost unanimously valued (M = 4.48–4.66), and no significant differences emerged, indicating shared recognition of the need for fully accessible environments for PwD. Psychological, emotional, and social well-being scored particularly high (for example, emotional well-being: M = 4.62, SD = 0.78). However, rugby participants reported lower values in sense of belonging (Z = –4.204, *p* < .001), peer support (Z = –2.327, *p* = .020), self-esteem (Z = –2.266, *p* = .023), and participation in decision-making (Z = –3.067, *p* = .002), suggesting a weaker perception of integration and empowerment. Relational, institutional, and rights-based items were also positively assessed, especially regarding trust, social relationships, and respect for rights. Nonetheless, significant differences appeared in sports insurance (Z = –3.811, *p* < .001), federation licensing (Z = –2.696, *p* = .007), membership fees (Z = –5.354, *p* < .001), and trust between athletes and clubs (Z = –2.710, *p* = .007), revealing rugby's more critical stance toward administrative barriers.

Concerning societal perceptions of how society could improve the lives of PwD (Table 4), rugby participants gave higher ratings in all items, emphasising support (Z = –2.146, *p* = .032), self-determination (Z = –2.533, *p* = .011), accessibility (Z = –2.906, *p* = .004), and education for inclusion (Z = –3.608, *p* < .001).

**Table 4:** Mann–Whitney *U* test results between basketball and rugby on how society can improve the lives of persons with disabilities.

Variable/Item	Mean Rank Basketball	Mean Rank Rugby	Mann–Whitney U	*Z*	*p*
By Providing the Necessary Support	112,66	125,84	6231,5	−2,146	.032
By Fostering Self-Determination	111,10	127,53	6039,0	−2,533	.011
By Creating Environments for Everyone and Removing Barriers	110,61	126,94	5992,0	−2,906	.004
By Educating Society About Diversity and the Need for Inclusion	110,20	128,50	5928,5	−3,608	.000

Table adapted from da-Silva (22). Author's elaboration.

Finally, contingency analyses showed no significant associations between years of experience and disability perceptions (Table 5), though trends indicated an association between years of experience and disability models, with athletes with longer experience more frequently aligning with social or rights-based perspectives (Φ = 0.245; Cramer's V = 0.173; *p* = .088). However, these results should be interpreted cautiously, as they do not indicate causal relationships. Most athletes (*N* = 153) aligned with a social or rights-based model, compared with a smaller group adopting a medical or rehabilitative perspective (*N* = 51). Moreover, participants with less than one year of experience were more likely to view disability as illness (residual = 2.0). Regarding the variable related to the impact of MA on perceptions of disability, participants with less than one year of experience presented a higher proportion of negative responses (residual = 1.8). Furthermore, for the general perception of disability variable, participants with less than one year of experience tended to identify more with the medical or rehabilitative model (residual = 1.3), while those with three to five years of experience were more frequently associated with the social or rights-based model (residual = 1.5).

**Table 5:** Measures of association in contingency tables between variables and years of experience in basketball and rugby.

Crossed Variables	*N*	Φ	Cramer's V	*p*
Belief about disability * Years of experience in MA	230	0.245	0.173	0.088
Change in perception of disability due to basketball * Years of experience in MA	236	0.177	0.125	0.498
General perception of disability * Years of experience in MA	208	0.186	0.131	0.128

Author's elaboration.

## 4 Discussion

This study analysed the implementation of the MA model in basketball, compared it with prior experience in rugby, and assessed its influence on disability perception. The comparison with rugby, a pioneering MA sport, allowed the identification of key differences, offering insights into the developmental and institutional maturity of the model across sporting contexts. The results indicated a highly positive evaluation of the MA model in basketball, encompassing formative, relational, intrapersonal, and structural factors, with mean scores above 4 out of 5. Similar findings were observed in rugby (22). Both sports showed convergence in valuing intrapersonal factors such as well-being, self-esteem, belonging, and inclusion, alongside interpersonal aspects linked to rights, trust, and relationships—elements that represented fundamental components for meaningful participation in inclusive programmes (31). The absence of belonging, low self-esteem, limited decision-making, and weak social networks had been shown to hinder inclusive sport sustainability (11, 32). Consistent with this evidence, participants viewed these elements as essential for both sports (22).

This convergence reinforced that inclusion extended beyond physical participation, involving social bonds, autonomy, and collective well-being (6). In this regard, the MA model promoted both formal and experiential inclusion, reflected in belonging and self-efficacy (23, 27, 28). Similar outcomes had been observed in other inclusive sport contexts (33, 34). According to Haegele et al. (35), PwD considered participation in mainstream sporting environments crucial, as it allowed them to feel part of a community and strengthen their sense of belonging. Therefore, mixed activities fostered reciprocity, social learning, and the development of empathy towards difference (36). However, comparisons revealed significant differences in intrapersonal indicators (belonging, support, self-esteem, decision-making) and interpersonal factors (insurance, licences, fees, institutional trust). This suggested that, despite the inclusive potential of the model, the culture and tradition of each sport modulated the intensity of its effects. In rugby, the MA model benefited from greater tradition and structural support, which might have explained the maturity of perceptions and the consolidation of community support networks (23).

The implementation of inclusive practices required time and collaboration among stakeholders to achieve systemic transformation (37). Sports federations and disability associations played key roles in embedding inclusive practices and supports (38). Therefore, progress depends on alignment with the Shared Citizenship Paradigm and the Quality of Life and Supports Model, ensuring coordinated implementation and sustainability across systems (37, 39). Previous research on MA rugby had shown that this approach contributed to positive changes in PwD's quality of life over time (11, 28). Similarly, participation in inclusive sporting activities had been associated with a more positive perception of quality of life, reflected in greater independence, improved social relationships, and enhanced overall well-being (40). In this study, significant differences emerged between sports concerning how society was perceived to contribute to improving PwD's quality of life. Results revealed that rugby participants emphasised, to a greater extent, the provision of adequate supports, the promotion of self-determination, the creation of accessible and equitable environments, and education fostering social awareness of diversity and inclusion.

Evidence suggested that education was essential in transforming attitudes towards disability, shifting from medical-rehabilitative to social and rights-based perspectives (41, 42). The results reflected the value of training to consolidate inclusion, not focused on MA rules but on diversity, natural supports, accessibility, and self-determination (22). In this sense, the formative dimension reflected growing awareness of the need to train different sporting agents in inclusion. The findings indicated that rugby participants assigned greater importance to specific training directed at coaches, referees, and support staff for model development compared to basketball. In this regard, communication constituted one of the main barriers to inclusive sport, affecting both families and technical staff, who often felt insecure when forming inclusive teams (43). Therefore, training should have promoted clear, respectful language and avoided paternalism, recognising athletes with disabilities as autonomous adults.

Perceptions of disability within MA environments tended to improve over time (11, 27). Most participants viewed disability through a social and rights-based lens aligned with MA philosophy, which recognised all athletes as equal citizens. Armila et al. (44), emphasised that understanding sport through this perspective enabled it to transcend rehabilitative behaviours and focus on its social value. Although contingency analyses did not reveal statistically significant associations between years of experience and perceptions of disability, descriptive patterns suggested an association between longer involvement in MA environments and a greater alignment with social and rights-based perspectives. However, these findings cannot be interpreted causally. It is possible that individuals who already hold more inclusive views of disability are more likely to engage with or remain in MA programmes over time, or that other factors such as prior training, personal values, or the inclusive culture of specific clubs may account for the observed pattern. This pattern aligns with findings from Abellán et al. (41) and Asbjørnslett and Bekken (36), who argued that contact and shared experiences with PwD fostered attitudinal changes and a more inclusive understanding grounded in equality and human rights. Accordingly, as emphasised throughout the study, values such as social inclusion and self-determination were crucial for guiding public policies and best practices aimed at individuals in situations of vulnerability and/or social exclusion. These principles are essential for transforming traditional medical and social models, promoting a more ethical, participatory, and inclusive organisational culture (45).

Based on the findings, several research avenues emerge to deepen understanding of the MA model's impact and sustainability across diverse sporting and sociocultural contexts. Future research should also explore model implementation in other sports beyond rugby and basketball, to identify facilitating factors and specific barriers influencing its transferability and institutional consolidation. Comparative analysis across countries and sporting cultures could yield valuable insights into the influence of support systems, inclusive policies, and federative structures on the model's effectiveness. Finally, further investigation is warranted into institutional policies that either facilitate or hinder large-scale implementation of the MA model.

## 5 Limitations

Despite the contributions of this study, several limitations had to be considered when interpreting the results. The cross-sectional design limited causal inference between the variables analysed, restricting understanding of how disability perceptions evolved over time. The non-probabilistic sample, limited to the only club developing Mixed Ability basketball in Spain at the time of data collection, constrains the generalisability of the findings to other contexts or sports. In contrast, the rugby data were collected from multiple clubs across different countries, which may limit the direct comparability of the results. Finally, potential social desirability bias and participant self-selection could have influenced some responses towards more favourable evaluations of the model, despite measures to ensure anonymity and confidentiality.

## 6 Conclusion

The MA model applied to basketball emerged as an effective strategy for promoting sporting, social, and emotional inclusion, with high evaluations across formative, structural, intrapersonal, and interpersonal factors. The results highlighted the roles of well-being, self-esteem, accessibility, and belonging as essential elements for the model's success.

Although differences were observed with rugby, both contexts agreed on the importance of strengthening the training of sports agents and enhancing the model's visibility to ensure sustainability. This reflected a favourable perception towards the consolidation and long-term viability of MA as a pathway to inclusive sporting participation. Thus, the MA model acted as a pedagogical catalyst, facilitating the transition from assistance-based conceptions to models centred on active citizenship and the recognition of rights.

## Data Availability

The raw data supporting the conclusions of this article will be made available by the authors, without undue reservation.
