# Supplementary material for: From rugby to basketball: a comparative analysis on the implementation of mixed ability
Source: Front Sports Act Living. 2026 Mar 16;8:1769269. doi: 10.3389/fspor.2026.1769269 (PMC13033746; doi:10.3389/fspor.2026.1769269)
Supplement: Supplementary file 1 [file Datasheet1.zip › Supplementary_Material_Figure 1.pdf]

Supplementary Material

1.1 Supplementary Figures

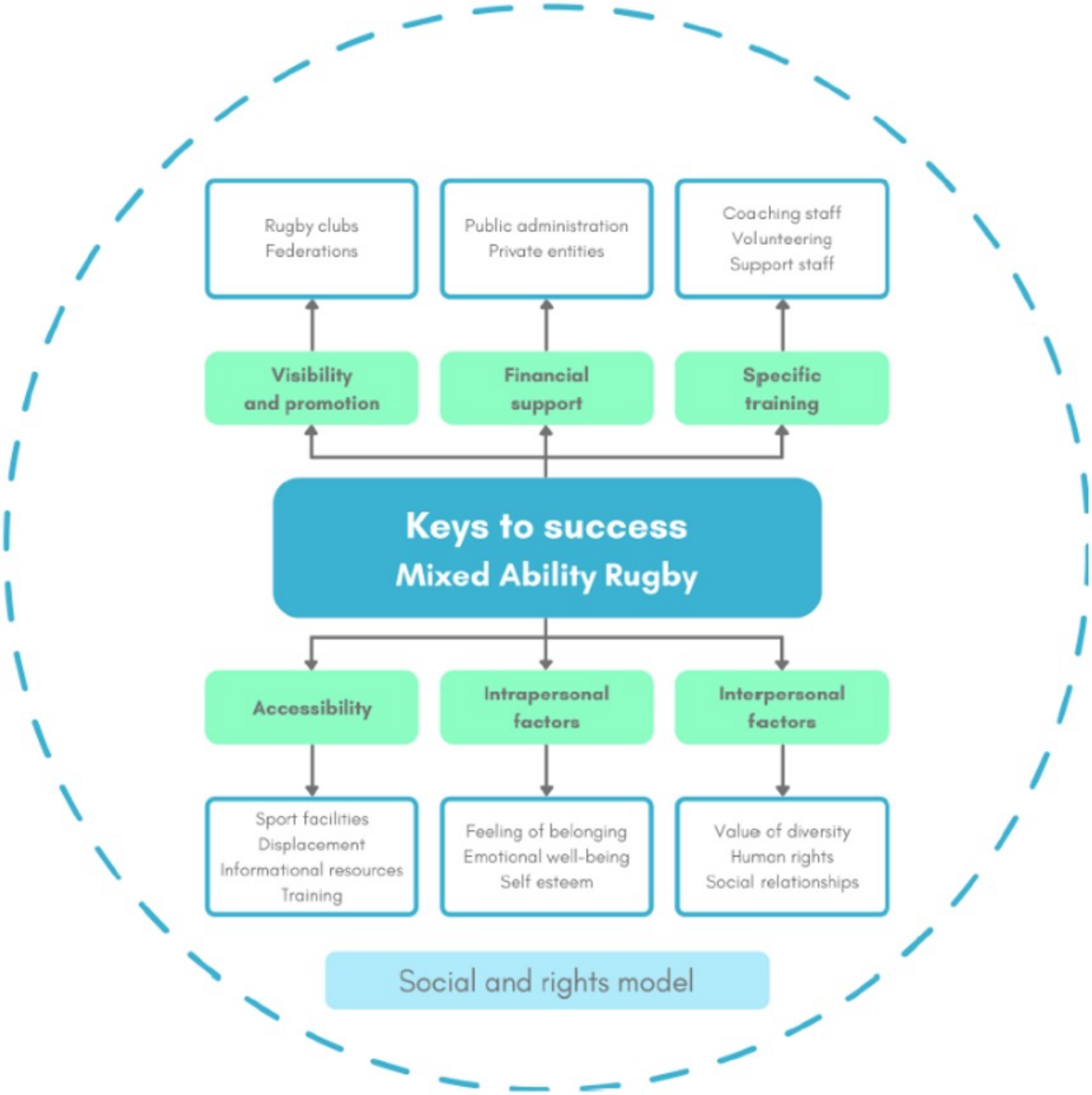

Supplementary Figure 1. Framework of the keys to success in Mixed Ability rugby.
Note. Adapted from da-Silva (2022). Author's elaboration.
