# Supplementary material for: From rugby to basketball: a comparative analysis on the implementation of mixed ability
Source: Front Sports Act Living. 2026 Mar 16;8:1769269. doi: 10.3389/fspor.2026.1769269 (PMC13033746; doi:10.3389/fspor.2026.1769269)
Supplement: Supplementary file 1 [file Datasheet1.zip › Supplementary_Material_T1.docx]

Supplementary Material

# Supplementary table 1.

**Table 1.** Sociodemographic characteristics of participants in MA rugby and basketball

| **Variable** | **Category** | **Rugby (%)** | **Basketball (%)** |
| --- | --- | --- | --- |
| **Gender** | Men | 64.9 | 48 |
|  | Women | 34.2 | 51.2 |
|  | Other | 0.9 | 0.8 |
| **Country of participation** | Spain | 67.5 | 100 |
|  | Argentina | 18.4 | 0 |
|  | Ecuador | 8.8 | 0 |
|  | Chile | 5.3 | 0 |
| **Role** | Player | 34.2 | 39 |
|  | Family member | 26.3 | 40.7 |
|  | Technical staff | 32.5 | 14.6 |
|  | Referee | 7 | 5.7 |
| **Years of experience** | < 1 year | 15.8 | 17.2 |
|  | 1 to 3 years | 36.8 | 29.5 |
|  | 3 to 5 years | 28.1 | 36.1 |
|  | 5 to 7 years | 13.2 | 17.3 |
|  | > 7 years | 6.1 | 0 |
| **Age** | Mean | 43.36 | 44.0 |

***Note.*** Table adapted from da-Silva (2022). Author's elaboration.
