# Supplementary material for: From rugby to basketball: a comparative analysis on the implementation of mixed ability
Source: Front Sports Act Living. 2026 Mar 16;8:1769269. doi: 10.3389/fspor.2026.1769269 (PMC13033746; doi:10.3389/fspor.2026.1769269)
Supplement: Supplementary file 1 [file Datasheet1.zip › Supplementary_Material_T2.docx]

Supplementary Material

# Supplementary table 2.

**Table 2.** Descriptive Statistics by Thematic Blocks of Mixed Ability Basketball and Rugby.

| **Thematic Block** | **Items** | **Sport** | **M** | **SD** | **Minimum** | **Maximum** | **N** |
| --- | --- | --- | --- | --- | --- | --- | --- |
| Visibility | 5a–5g | Baketball | 4.49 | 0.88 | 1 | 5 | 123 |
|  |  | Rugby | 4.69 | 0.66 |  |  | 114 |
| Promotion of Specific Plans | 6a–6g | Baketball | 4.40 | 0.96 | 1 | 5 | 123 |
|  |  | Rugby | 4.57 | 0.78 |  |  | 114 |
| Financial Support | 7a–7g | Baketball | 4.40 | 0.97 | 1 | 5 | 123 |
|  |  | Rugby | 4.39 | 0.91 |  |  | 114 |
| Training | 8a–8g | Baketball | 4.39 | 0.98 | 1 | 5 | 123 |
|  |  | Rugby | 4.65 | 0.67 |  |  | 114 |
| Accessibility | 9a–9e | Baketball | 4.57 | 0.84 | 1 | 5 | 123 |
|  |  | Rugby | 4.59 | 0.75 |  |  | 114 |
| Intrapersonal Factors | 10a–10i | Baketball | 4.54 | 0.82 | 1 | 5 | 123 |
|  |  |  | 4.73 | 0.55 |  |  |  |
|  |  | Rugby |  |  |  |  | 114 |
| Interpersonal Factors | 11a–11h | Baketball | 4.38 | 0.90 | 1 | 5 | 123 |
|  |  | Rugby | 4.70 | 0.57 |  |  | 114 |

***Note.*** Table adapted from da-Silva (2022). M = Mean; SD = Standard deviation. Author's elaboration.
