# Supplementary material for: From rugby to basketball: a comparative analysis on the implementation of mixed ability
Source: Front Sports Act Living. 2026 Mar 16;8:1769269. doi: 10.3389/fspor.2026.1769269 (PMC13033746; doi:10.3389/fspor.2026.1769269)
Supplement: Supplementary file 1 [file Datasheet1.zip › Supplementary_Material_T3.docx]

Supplementary Material

# Supplementary table 3.

**Table 3.** Mann–Whitney U Test Results Between Basketball and Rugby for the Different Thematic Blocks

| **Variable / Item** | **Mean Rank Basketball** | **Mean Rank Rugby** | **Mann–Whitney U** | **Z** | **p** |
| --- | --- | --- | --- | --- | --- |
| Visibility – Sports Clubs (5c) | 108,84 | 129,96 | 5761,5 | -3,439 | .001 |
| Visibility – Formal Education (5e) | 112,14 | 126,40 | 6167,5 | -2,013 | .044 |
| Promotion of Specific Plans – Sports Clubs (6c) | 110,37 | 128,32 | 5949,0 | -2,788 | .005 |
| Training – Coaching Staff (8b) | 111,67 | 126,91 | 6109,5 | -2,384 | .017 |
| Training – Referees (8c) | 109,01 | 128,66 | 5796,0 | -2.765 | .006 |
| Training – Support Staff (8f) | 112,13 | 126,41 | 6166,0 | -2,172 | .030 |
| Sense of Belonging to the Club (10a) | 105,63 | 133,43 | 5366,0 | -4,204 | .000 |
| Support from Other Categories (10b) | 111,18 | 127,44 | 6049,0 | -2.327 | .020 |
| Improvement in Self-Esteem (10e) | 112,26 | 126,27 | 6182,0 | -2,266 | .023 |
| Opportunity to Participate in Decision-Making (10i) | 109,80 | 128,92 | 5880,0 | -3.067 | .002 |
| Sports Insurance (11a) | 106,33 | 132,67 | 5452,5 | -3,811 | .000 |
| Federation Licence (11b) | 108,43 | 130,41 | 5710,5 | -2,696 | ,007 |
| Membership Fee (11c) | 97,19 | 142,53 | 4328,5 | -5,354 | .000 |
| Trust Between Athletes and Club (11d) | 110,62 | 128,04 | 5980,0 | -2,710 | .007 |

***Note.*** Table adapted from da-Silva (2022). Author's elaboration
