# Supplementary material for: From rugby to basketball: a comparative analysis on the implementation of mixed ability
Source: Front Sports Act Living. 2026 Mar 16;8:1769269. doi: 10.3389/fspor.2026.1769269 (PMC13033746; doi:10.3389/fspor.2026.1769269)
Supplement: Supplementary file 1 [file Datasheet1.zip › Supplementary_Material_T4.docx]

Supplementary Material

# Supplementary table 4.

**Table 4.** Mann–Whitney U Test Results Between Basketball and Rugby on how Society can Improve the Lives of Persons with Disabilities

| **Variable / Item** | **Mean Rank Basketball** | **Mean Rank Rugby** | **Mann–Whitney U** | **Z** | **p** |
| --- | --- | --- | --- | --- | --- |
| By Providing the Necessary Support | 112,66 | 125,84 | 6231,5 | -2,146 | . 032 |
| By Fostering Self-Determination | 111,10 | 127,53 | 6039,0 | -2,533 | .011 |
| By Creating Environments for Everyone and Removing Barriers | 110,61 | 126,94 | 5992,0 | -2,906 | ,004 |
| By Educating Society About Diversity and the Need for Inclusion | 110,20 | 128,50 | 5928,5 | -3,608 | ,000 |

***Note.*** Table adapted from da-Silva (2022). Author's elaboration
