# Supplementary material for: From rugby to basketball: a comparative analysis on the implementation of mixed ability
Source: Front Sports Act Living. 2026 Mar 16;8:1769269. doi: 10.3389/fspor.2026.1769269 (PMC13033746; doi:10.3389/fspor.2026.1769269)
Supplement: Supplementary file 1 [file Datasheet1.zip › Supplementary_Material_T5.docx]

Supplementary Material

# Supplementary table 5.

**Table 5.** Measures of Association in Contingency Tables Between Variables and Years of Experience in Basketball and Rugby

| **Crossed Variables** | **N** | **Φ** | **Cramer’s V** | **p** |
| --- | --- | --- | --- | --- |
| Belief about disability * Years of experience in MA | 230 | 0.245 | 0.173 | 0.088 |
| Change in perception of disability due to basketball * Years of experience in MA | 236 | 0.177 | 0.125 | 0.498 |
| General perception of disability * Years of experience in MA | 208 | 0.186 | 0.131 | 0.128 |

*Note:* Author's elaboration
